# Decompressive laparotomy for abdominal compartment syndrome – a critical analysis

**DOI:** 10.1186/cc4870

**Published:** 2006-03-27

**Authors:** Jan J De Waele, Eric AJ Hoste, Manu LNG Malbrain

**Affiliations:** 1 Intensive Care Unit, Ghent University Hospital, Gent, Belgium; 2 Intensive Care Unit, Campus Stuivenberg, ZiekenhuisNetwerk Antwerpen, Antwerp, Belgium

## Abstract

**Introduction:**

Abdominal compartment syndrome (ACS) is increasingly recognized in critically ill patients, and the deleterious effects of increased intraabdominal pressure (IAP) are well documented. Surgical decompression through a midline laparotomy or decompressive laparotomy remains the sole definite therapy for ACS, but the effect of decompressive laparotomy has not been studied in large patient series.

**Methods:**

We reviewed English literature from 1972 to 2004 for studies reporting the effects of decompressive laparotomy in patients with ACS. The effect of decompressive laparotomy on IAP, patient outcome and physiology were analysed.

**Results:**

Eighteen studies including 250 patients who underwent decompressive laparotomy could be included in the analysis. IAP was significantly lower after decompression (15.5 mmHg versus 34.6 mmHg before, *p* < 0.001), but intraabdominal hypertension persisted in the majority of the patients. Mortality in the whole group was 49.2% (123/250). The effect of decompressive laparotomy on organ function was not uniform, and in some studies no effect on organ function was found. Increased PaO_2_/FIO_2 _ratio (PaO_2 _= partial pressure of oxygen in arterial blood, FiO_2 _= fraction of inspired oxygen) and urinary output were the most pronounced effects of decompressive laparotomy.

**Conclusion:**

The effects of decompressive laparotomy have been poorly investigated, and only a small number of studies report its effect on parameters of organ function. Although IAP is consistently lower after decompression, mortality remains considerable. Recuperation of organ dysfunction after decompressive laparotomy for ACS is variable.

## Introduction

Intraabdominal hypertension (IAH) is a clearly identified cause of organ dysfunction in patients after emergency abdominal surgery and trauma [1-3]. It is also increasingly recognized in other patients in the intensive care unit (ICU), for example, after elective surgical procedures [4], liver transplantation [5], massive fluid resuscitation for extraabdominal trauma [6] and severe burns [7]. The presence of IAH at admission to the ICU has been associated with severe organ dysfunction during the ICU stay, and the development of IAH during ICU stay was an independent predictor of mortality [4].

The clinical picture resulting from sustained IAH has been described as abdominal compartment syndrome (ACS). Although understanding of the pathophysiology of IAH has greatly improved [8,9], few advances have been made in the treatment of ACS. Few non-surgical options are available for the treatment of ACS. In some patients, IAH is caused by intraperitoneal fluid, and in these patients percutaneous drainage may be an option, as has been described in patients with ACS after burns [10]. The use of gastric and rectal tubes to drain air and gastrointestinal contents has been proposed by experts, but a scientific foundation is lacking [11]. Other proposed therapies include ultrafiltration [12] and the use of muscular blocking agents [13].

Surgical decompression is the only available definite treatment for IAH, and numerous case series have been reported, but the effects of surgical decompression have not been reviewed in large series; patients who require decompression are frequently a selected subpopulation of the total study population. Also, most papers focus on factors associated with IAH and its effects, rather than specifically looking at endpoints, such as hospital mortality and organ function after surgical decompression.

The goal of this review is to describe the effect of surgical decompression through a midline laparotomy (termed 'decompressive laparotomy' (DL) in this review) on intraabdominal pressure (IAP) and the outcome and physiology of patients undergoing this procedure.

## Materials and methods

Relevant articles were identified through a computerized search of the English literature using Web of Science version 7.2 (ISI Thomson, Philadelphia, USA) for the years 1972 to 2004. Search terms included 'intraabdominal hypertension' OR 'abdominal compartment syndrome' and 'decompressive laparotomy' OR 'decompression'. Review articles, case reports and case series describing fewer than four patients were excluded from the analysis.

Articles describing adult patients with IAH requiring decompression were included in the analysis if: details on IAP – at least before decompression – were available; and the outcome was available for all patients who underwent abdominal decompression. In this setting, DL was defined as a surgical intervention on the abdominal wall aimed at reducing the IAP, after which a temporary abdominal closure device was used; percutaneous drainage of fluid collections or escharotomies were not considered in this review.

The bibliographies of the articles that were included in the final analysis were reviewed for relevant publications that would have been missed by the computerized search.

For the articles retrieved, we classified the ACS according to the current guidelines of the World Society of Abdominal Compartment Syndrome [14] (Table 1), and recorded the indication for decompression. The effect of abdominal decompression on organ function was recorded; hemodynamic (blood pressure, heart rate, cardiac output, central venous pressure, pulmonary occlusion pressure, systemic vascular resistance and oxygen delivery indices), ventilatory (PaO_2_/FIO_2 _ratio (PaO_2 _= partial pressure of oxygen in arterial blood, FiO_2 _= fraction of inspired oxygen), peak airway pressure, lung compliance expressed by static or dynamic compliance) and renal function parameters (urinary output) were retrieved. Patient characteristics such as age, disease severity as expressed by the Acute Physiology and Chronic Health Evaluation (APACHE) II score or Injury Severity score (ISS), and the timing of DL after the precipitating event (hospital admission or prior abdominal surgical intervention) were recorded when available.

Statistical analysis was performed using SPSS for Windows 12.0^® ^(SPSS, Chicago, IL, USA). IAP and physiological variables before and after DL were compared using paired samples *t* test. Continuous data are expressed as mean (standard deviation). A double sided *p* value of less than 0.05 was considered statistically significant.

## Results

The computerized search yielded 85 papers, 19 of which could be included in the analysis based on the analysis of the type of article and review of the abstract. From the references in these articles, another 8 papers were considered to contain significant data, bringing the total number of studies reporting on patients who underwent surgical decompression to 27. After analysis of the data available in the papers, 9 papers were excluded because of various reasons (no data on IAP available (*n *= 5), no DL performed as a means of decompression (*n *= 1), analysis based on patients already described in another paper that was included in the analysis (*n *= 1), indication for laparotomy planned for reasons other than ACS (*n *= 1), and insufficient data on the groups of patients that were decompressed (*n *= 1).

The 18 papers included in the final analysis are in listed in Table 2. In total, 250 patients were treated with DL for ACS, of which 174 had primary ACS and 76 secondary ACS.

In four papers no indication for DL was named, but it could be presumed it was ACS. No clear definition of ACS was mentioned in another five papers, and only six used a more or less clear definition of ACS, including a cut off IAP level (Table 2). The definitions of ACS were different in every paper, and most noticeably the critical level of IAP that was considered an indication for DL varied from 18 to 30 mmHg. In one paper, uncontrollable intracranial pressure was the sole indication for DL [15]. Mean interval from admission to the hospital or from the previous surgical intervention to DL was reported only in a limited number of papers, and varied from 12 to 38 hours, except from the study in which uncontrollable ICP was the indication for DL; in this paper, the mean interval between admission and DL was 139 hours.

### Effect of surgical decompression on IAP

**Figure 1:**
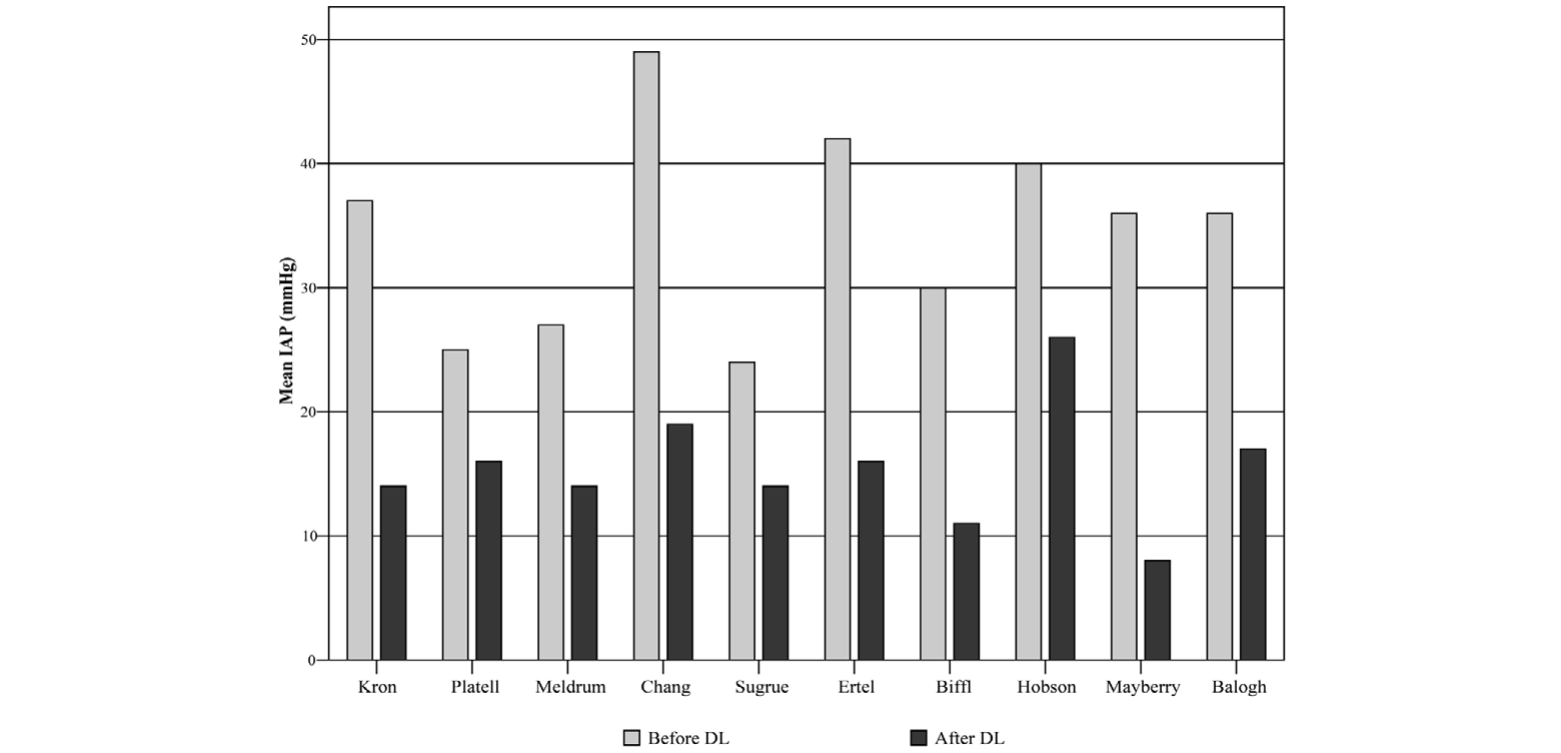
The effect of decompressive laparotomy (DL) on intraabdominal pressure (IAP) in patients with primary and secondary abdominal compartment syndrome. IAP levels are those reported in individual papers in the study; Kron and colleagues [23], Platell and colleagues [26], Meldrum and colleagues [17], Chang and colleagues [28], Sugrue and colleagues [18], Ertel and colleagues [2], Biffl and colleagues [32], Hobson and colleagues [16], Mayberry and colleagues [34], Balogh and colleagues [35].

From 10 studies, IAP values before and after abdominal decompression were available from a total of 161 patients; the other studies only reported IAP values before decompression. In all but one report [16], IAP fell significantly after surgical decompression (Figure 1). Overall, the mean reported IAP before DL was 34.6 mmHg (8.06) and fell to 15.5 mmHg (4.81) after DL (*p *< 0.001).

### Outcome after surgical decompression for ACS

Mortality rates for patients who underwent surgical decompression for ACS are summarized in Table 3. Overall, reported mortality for all patients with ACS who underwent surgical decompression was 49.2% (123/250). The mean age in the different studies was 44.5 years. The severity of disease, as assessed by APACHE II score and ISS, is generally high in these patients, but was not available in most of the papers; an APACHE II-based predicted mortality, therefore, could not be calculated for these patients.

The cause of death of patients who underwent DL could be retrieved from only nine of the studies. This accounted for only 29 out of the total of 123 patients who died. The main cause of death after DL was single or multiple organ dysfunction (*n *= 23, 79%); other causes included head injury (*n *= 2, 7%) and haemorrhage (*n *= 1, 3%). In three patients, therapy was withdrawn.

### Effect of abdominal decompression on hemodynamic, respiratory and renal function parameters

Table 4 summarizes the effect of abdominal decompression on hemodynamic physiological variables considered to be impaired because of ACS. Blood pressure remained unchanged after decompression in five out of nine reports, but increased significantly in the remainder. A significant drop in central venous pressure was present in three out of eight papers, and four out of eight reported a significantly lower pulmonary artery occlusion pressure. Heart rate was found to be unchanged in all but two reports. In the majority of the papers that studied cardiac function before and after decompression, the cardiac output or cardiac index improved significantly after decompression.

A small number of studies reported detailed information on hemodynamic parameters: one study found an increased oxygen delivery after decompression, whereas another found no difference. Systemic vascular resistance decreased in two studies, but increased in one. No differences in SvO_2 _(mixed venous oxygen saturation) were found in both studies reporting details on this topic.

The effect of DL on respiratory function is presented in Table 5. In all studies, respiratory function improved significantly in most patients, as well as in terms of reduced peak inspiratory pressures and improved PaO_2_/FIO_2 _ratio. In all reports, PaO_2_/FIO_2 _ratios after decompression remained below 300, ranging from 154 to 239.

**Figure 2:**
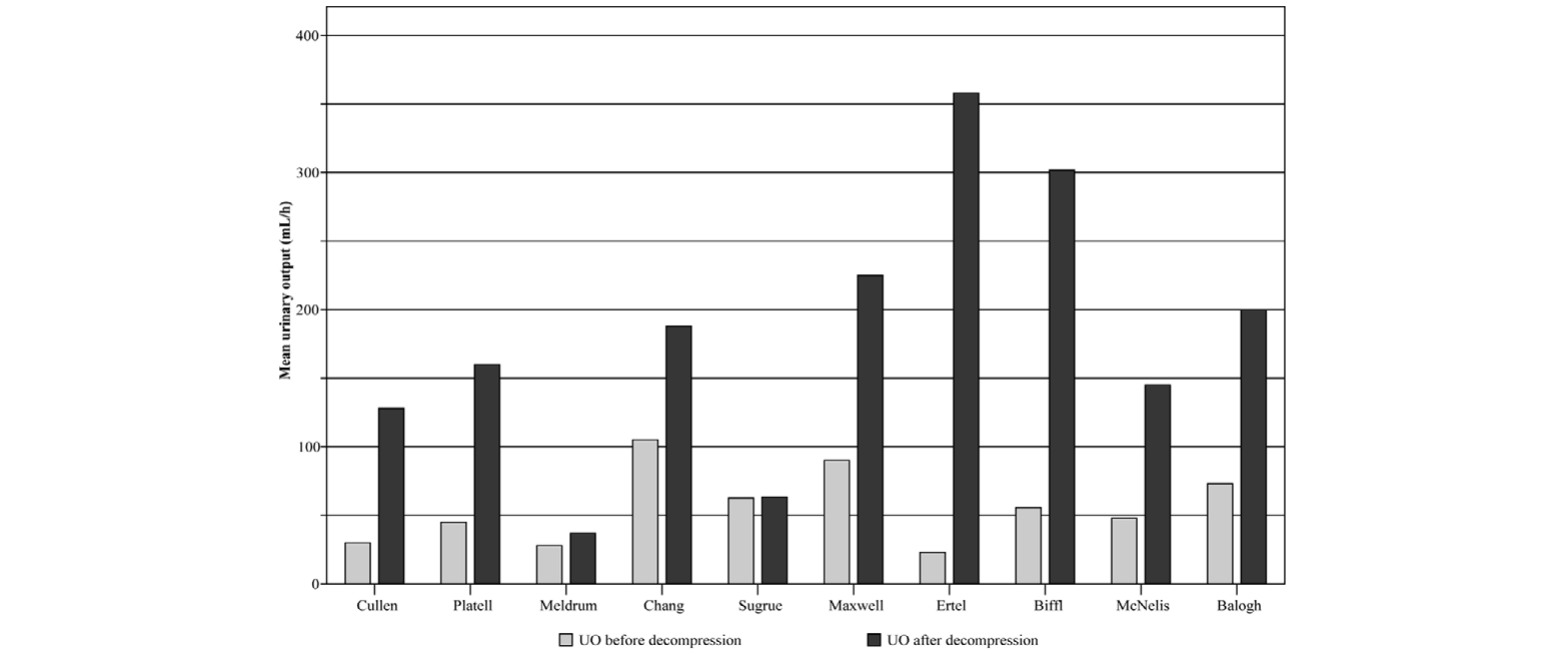
Effect of decompressive laparotomy on urinary output in 10 studies reporting urinary output (UO) before and after decompressive laparotomy. Data are from individual papers in this study; Cullen and colleagues [25], Platell and colleagues [26], Meldrum and colleagues [17], Chang and colleagues [28], Sugrue and colleagues [18], Maxwell and colleagues [29], Ertel and colleagues [2], Biffl and colleagues [32], McNelis and colleagues [33], Balogh and colleagues [35].

In two of the larger patient series [17,18], there was no change in urinary output (Figure 2). In papers that reported a limited number of patients, absolute values increased, but the number of patients is probably too limited to reach statistical significance. In 5 out of 10 studies, the mean urinary output was above 50 ml/hour before decompression (mean urinary output ranged from 50 ml/hour to 105 ml/hour) and, in most of these, it significantly increased after decompression.

## Discussion

DL resulted in a decrease in IAP in all patients who were studied. However, IAH persisted in a considerable number of patients, as the mean IAP after DL remained well above the 12 mmHg threshold for IAH. In one study, the IAP after decompression was as high as 26 mmHg. The fact that IAP decreased is of course not surprising, but the level of IAP after surgical intervention is more intriguing. Apparently, several patients must have suffered from (early) recurrent or persistent ACS in these studies, although only a few studies specifically mention this problem.

Important limitations here are the facts that almost half of the studies (accounting for about a third of the patients in this review) did not report IAP values after DL and that the time to measurement of IAP after DL was not standardized. The problem of recurrent ACS in patients with open abdomen treatment has been reported by Gracias and colleagues [19]. Mortality in their patients with recurrent ACS was high when compared to patients without recurrent ACS (60% versus 7%); recurrent ACS occurred between 1.5 and 12 hours after surgery. From the data available, it is not clear whether recurrent ACS is an independent risk factor for mortality, but considering the association of organ dysfunction and mortality in recent epidemiological studies [4], it seems plausible that this is a major factor determining outcome in these patients.

Mortality in patients undergoing DL remains high and deserves further investigation. Several factors may explain the fact that half of the patients in the included studies eventually died, in spite of aggressive measures like DL. First of all, patients who require DL are severely ill at the moment of decompression, and often DL is considered a last resort. This may not be reflected by APACHE II scores early after ICU admission or the ISS, although in the few studies that reported these parameters, these were high to very high. Obviously, as no control group is available, it is difficult to guess the outcome of these patients without decompression.

Secondly, the fact that IAP remained moderately to severely elevated in a number of patients (who can be considered incomplete or non-responders) should also be taken into account. This is also reflected by the fact that although a number of physiological values improved, these did not return to normal. The effect of DL on oxygenation is one such example. The mean PaO_2_/FIO_2 _ratio after decompression remained far below 300 in all the reports, and below 200 in most of them, notably in the two largest studies [6,18]. Unfortunately, no data on the effect on organ dysfunction as assessed by serial scoring systems designed to study the evolution of organ dysfunction, such as the SOFA score, are available. Moreover, from the variables included in the SOFA score, only one out of six organ systems (the respiratory system) could be graded by the parameters reported in the studies in this review. The data reported for the cardiovascular, haematological, renal, neurological and gastrointestinal systems are incomplete or lacking in most studies. Although the parameters most notably impaired by the development of ACS, such as peak inspiratory pressure, mean arterial pressure and urinary output, are often significantly improved, these might not be the best parameters for studying organ function. To evaluate the cardiovascular system, information on the amount of vasoactive medications should be mentioned; serum creatinine probably should be included to evaluate renal function.

Thirdly, it should be considered that DL may be harmful for some patients. Morris and colleagues [20] described a lethal reperfusion syndrome early after DL. There may be a risk of re-bleeding when coagulation is not completely restored before considering abdominal decompression, especially in trauma patients who are often severely coagulopathic early after arrival in the ICU. Hemorrhagic shock was the cause of death in a third of the deaths after DL in the paper by Ertel and colleagues [2]; Balogh and colleagues [1] reported that exsanguination was the cause of death in two out of six patients with secondary ACS who were decompressed and later died. Also, in patients with severe acute pancreatitis and ACS, we found that three out of four patients who were decompressed died, two of them from uncontrollable haemorrhage [21].

Although DL has a positive effect on cardiovascular, respiratory and renal function, some issues require further investigation. Filling pressures (central venous pressure and pulmonary artery pressure) decreased in all patients, but this probably only reflects the decrease in IAP in those patients. It has been shown that IAP is transduced to a large extent (25% to 80%) to the thoracic cage [22], resulting in the high central venous and pulmonary occlusion pressures often observed in ACS. The decrease after decompression does not necessarily reflect an improvement in organ function. Cardiac function improved in the majority of the patients, but it is remarkable that in the largest study no improvement in cardiac index was found. The change in peak airway pressure is not surprising. Some of the studies date from the era when normal tidal volumes (8 to 12 ml/kg) were used to ventilate patients with acute respiratory distress syndrome, so the decrease in peak airway pressure and improvement in compliance may be more pronounced than when lower tidal volumes are used. The effect on oxygenation was positive overall, but respiratory function remained severely impaired in the majority of the patients. There was no change in urinary output in the two largest series and, remarkably, the urinary output before DL in patients with ACS was about 50 ml/hour or more in the majority of the papers. Nevertheless, significant improvement was found in all but two papers, often despite the small number of patients. Sugrue and colleagues [18] reported an increase in serum creatinine after DL with only little improvement over 14 days. No data on short or long term effects of renal function were reported in the other papers.

Some questions cannot be answered by the analysis of the outcome parameters in this review. The effects of the timing of DL and the speed of diagnosis of ACS on patient outcome remain to be elucidated. The timing of surgical intervention was only rarely reported, and it is not clear from the papers presented which clinical condition exactly triggered surgical decompression in the patients reported. Also, coexisting causes of organ dysfunction, such as sepsis or acute respiratory distress syndrome, in these severely ill patients and its role in the development of IAH and ACS should be further explored. Patients suffering from severe sepsis often have increased fluid requirements, which in itself may contribute to recurrent ACS [6].

Although there is a consensus on the definition of ACS, there is no clear consensus for which parameter should be the threshold for surgical decompression in patients with ACS; no clear conclusion can be drawn from this review either. Several authors have suggested that an IAP of more than 25 should trigger DL [17,23]. Others suggest that the IAP recordings are only supportive data, and base the decision to open the abdomen on clinical parameters [24]. The clinical condition of the patient with secondary ACS makes the whole picture often very complicated. Often, these patients have other causes of hypotension, renal dysfunction or respiratory failure, and the development of IAH may be a factor contributing to the clinical picture of ACS. This concern was also raised by Balogh and colleagues [1], who considered ACS to be an indicator of disease severity, not the cause of early death.

## Conclusion

Patients with primary and secondary ACS generally are good responders to DL in terms of reduction of IAP and improvement of several physiological variables, but the exact effect on organ dysfunction is not clear. An important next step in the management of patients with primary and secondary ACS is to identify those patients who would benefit most from DL, as this review indicates that recuperation of organ dysfunction is variable and unpredictable, and mortality remains considerable in patients treated with it. In both primary and secondary ACS, the IAP value probably is not the only parameter that should be considered and clinical parameters should be included when evaluating a patient with IAH for surgical decompression.

To study the effect of abdominal decompression in a larger series of patients, we propose to open a registry of patients with ACS undergoing abdominal decompression, coordinated by the World Society of Abdominal Compartment Syndrome (WSACS); more information can be found at the society's website [14].

## Key messages

• 	Detailed effects of DL on organ function are only rarely reported.

• 	IAP threshold levels for DL reported in the literature vary considerable.

• 	DL decreases IAP to levels below 20 mmHg in most studies.

• 	A positive effect on organ function is reported in most studies, but the effect is inconsistent, and the duration of this effect is not clear.

• 	Reported mortality after DL for ACS is high.

## Abbreviations

ACS = abdominal compartment syndrome; APACHE = Acute Physiology and Chronic Health Evaluation; CI=cardiac index; CVP = central venous pressure; DL = decompressive laparotomy; DO2I = Oxygen delivery index; HR = heart rate; IAH = intraabdominal hypertension; IAP = intraabdominal pressure; ICP = intracranial pressure; ICU = intensive care unit; ISS = Injury Severity score; MAP = mean arterial pressure; NA = not available; SOFA = sepsis related organ failure assessment; SVRi = systemic vascular resistance index.

## Competing interests

JDW and EH declare that they have no competing interests. MM is a member of the medical advisory board of Pulsion Medical Systems (owns 100 Pulsion shares) and is president of the World Society for Abdominal Compartment Syndrome

## Authors' contributions

JDW conceived and designed the study, acquired a substantial portion of the data, analysed and interpreted the data, drafted the manuscript, provided statistical expertise and supervised the study (taking overall responsibility for all aspects of it).

EH and MM analysed and interpreted data and critically revised the manuscript for important intellectual content.

## Figures and Tables

**Table 1:** Consensus definitions of intraabdominal hypertension, and abdominal compartment syndrome (primary, secondary and recurrent) according to WSACS

Term	Definition
IAH	IAH is defined by a sustained increase in IAP of 12 mmHg or more, recorded by a minimum of three standardized measurements conducted 4 to 6 hours apart, with or without an APP <60 mmHg
ACS	ACS is defined as a sustained increase in IAP of 20 mmHg or more with or without APP <60 AND single or multiple organ system failure that was not previously present
Primary ACS	ACS caused by:
	A condition associated with injury or disease in the abdomino-pelvic region that frequently requires early surgical or angioradiological intervention, OR
	A condition that develops following abdominal surgery (such as abdominal organ injuries that require surgical repair or damage control surgery, secondary peritonitis, bleeding pelvic fractures or other cause of massive retroperitoneal haematoma, liver transplantation)
Secondary ACS	ACS caused by conditions that do not originate from the abdomen (such as sepsis and capillary leak, major burns, and other conditions requiring massive fluid resuscitation), yet result in the signs and symptoms commonly associated with primary ACS
Recurrent ACS	ACS caused by a condition in which it develops following prophylactic or therapeutic surgical or medical treatment of primary or secondary ACS (e.g., persistence of ACS after DL or development of a new ACS episode following definitive closure of the abdominal wall after the previous utilization of a temporary abdominal wall closure)

ACS, abdominal compartment syndrome; APP, abdominal perfusion pressure; DL, decompressive laparotomy; IAH, intraabdominal hypertension; WSACS, World Society of Abdominal Compartment Syndrome.

**Table 2:** Overview of 18 papers included in the final analysis

Reference	Journal	Year	No. of patients			Indication for abdominal decompression	Delay to decompression (hours)
			Total	Primary ACS	Secondary ACS		

[23]	*Ann Surg*	1984	4	4	0	IAP >25 + acute renal failure	NA
[25]	*Crit Care Med*	1989	6	6	0	NA (presumably ACS)	NA
[26]	*Aust NZ Surg*	1990	10	10	0	IAP >18 + organ dysfunction	NA
[27]	*S Afr Med J*	1995	4	3	1	ACS	16
[17]	*Am J Surg*	1997	21	21	0	ACS	27
[28]	*J Trauma*	1998	11	11	0	IAP >25 mmHg + organ dysfunction	38
[18]	*J Trauma*	1998	49	49	0	ACS	18
[29]	*J Trauma*	1999	6	0	6	NA (presumably ACS)	NA
[2]	*Crit Care Med*	2000	17	13	4	ACS	13
[30]	*J Trauma*	2000	6	0	6	NA (presumably ACS)	NA
[31]	*Am J Surg*	2001	28	28	0	IAP >20 + organ dysfunction	17
[32]	*Am J Surg*	2001	10	0	10	ACS	12
[16]	*J Trauma*	2002	4	0	4	ACS unresponsive to conservative measures	NA
[10]	*J Burn Care Rehab*	2002	4	0	4	IAP >30 + renal or ventilatory impairment	28
[33]	*Am Surg*	2002	18	17	1	ACS	NA
[34]	*Arch Surg*	2003	9	0	9	NA (presumably ACS)	17
[35]	*J Trauma*	2003	26	11	15	IAP >25 mmHg + progressive organ dysfunction	13
[15]	*J Trauma*	2004	17	1	16	Uncontrollable ICP	139
Total			250	174	76		

ACS, abdominal compartment syndrome; IAP, intraabdominal pressure; ICP, intracranial pressure; NA, not available.

**Table 3:** Patient characteristics and outcome after decompressive laparotomy

Reference	Year	No. of patients included	Age (years)	ISS	APACHE II score	IAP before DL (mmHg)	IAP after DL (mmHg)	Mortality (%)
Primary ACS								
[23]	1984	4	55	-	-	37	14	50
[25]	1989	4	-	-	-	37	-	75
[26]	1990	10	69	-	-	25	16	60
[17]	1997	21	39	26	-	27	14	29
[28]	1998	11	37	27	-	49	19	64
[18]	1998	49	36	37	-	33	-	42
[31]	2001	28	36	32	-	26	-	43
Secondary ACS								
[29]	1999	6	57	-	27	24	14	67
[30]	2000	6	45	-	-	31	-	67
[32]	2001	10	45	-	-	30	11	50
[16]	2002	4	-	-	25	40	26^a^	75
[10]	2002	4	17	-	-	34	-	100
[34]	2003	9	47	24	-	36^a^	8^a^	22
Mixed primary and secondary ACS								
[27]	1995	4	-	-	-	39,5^a^	-	24
[2]	2000	17	42	37	21	42^a^	16^a^	65
[33]	2002	18	69	-	20	43	-	61
[35]	2003	26	-	28	-	36	17	28
[15]	2004	17	29	-	29,5	29,5	-	35
Mean (SD)			44 (14.4)	30 (5.3)	25 (4.0)	35.5 (8.06)	15.5 (4.81)	

^a^Data coming from incomplete datasets. dash, not available; ACS, abdominal compartment syndrome; APACHE II, Acute Physiology and Chronic Health Evaluation; DL, decompressive laparotomy; IAP, intraabdominal pressure; ISS, Injury Severity score; SD, standard deviation.

**Table 4:** Effect of decompressive laparotomy on hemodynamic variables reported in 13 studies

Reference	*n*	MAP		CVP		PAOP		CI		HR		SVRi		DO_2_I	
		Before	After	Before	After	Before	After	Before	After	Before	After	Before	After	Before	After

[25]	6	87	73	**26**	**15**	**27**	**17**	**4.5**^a^	**6.4**^a^	121	122	-	-	-	-
[27]	4	99.25	133	18.5	12.5	-	-	-	-	-	-	-	-	-	-
[17]	21	-	-	16	14	**19**	**14**	**3.3**	**3.9**	-	-	**1,187**^b^	**842**^b^	**542**	**633**
[28]	11	-	-	-	-	**30**	**24**	3.7	3.9	**124**	**107**	**1,634**	**1,874**	-	-
[18]	49	93.5	97,6	-	-	-	-	-	-	116	111	-	-	-	-
[29]	6	**126**^c^	**146**^c^	-	-	11	11	-	-	-	-	-	-	-	-
[2]	17	94	81	**21**	**15**	**32**	**18**	**4.8**	**7**	**128**	**105**	-	-	-	-
[32]	10	**102**^c^	**124**^c^	17.3	17.5	18.8	17.8	3.5	2.9	124	121	-	-	-	-
[16]	4	75	77	24	21	-	-	-	-	-	-	-	-	-	-
[33]	18	-	-	-	-	19.5	16.4	**2.8**	**3.2**	-	-	-	-	-	-
[34]	9	**100**^c^	**125**^c^	**22**	**14**	-	-	-	-	-	-	-	-	-	-
[35]	26	**79**	**92**	20	18	18	17	**3.1**	**3.8**	-	-	**2,362**	**1,524**	469	517
[15]	17	-	-	20.9	18.9	-	-	4.6	4.7	-	-	-	-	-	-

^a^Cardiac output; ^b^systemic vascular resistance; ^c^systolic arterial pressure. Numbers in bold are the significant difference between value before and after decompression. CI, cardiac index; CVP, central venous pressure; DO_2_I, oxygen delivery index; HR, heart rate; MAP, mean arterial pressure; PAOP, pulmonary artery occlusion pressure; SVRi, systemic vascular resistance index.

**Table 5:** Effect of decompressive laparotomy on respiratory variables as reported in 14 studies

Reference	*n*	PaO_2_/FIO_2 _ratio		Peak airway pressure		Static compliance		Dynamic compliance	
		Before	After	Before	After	Before	After	Before	After

[25]	6	**120**	**239**	-	-	-	-	-	-
[27]	4	-	-	57.25	42.75	-	-	-	-
[17]	21	**177**	**229**	**44**	**32**	**27**	**38**	-	-
[28]	11	**165**	**236**	-	-	-	-	**13**	**24**
[18]	49	180	193	35.5	33.5	-	-	**24.1**	**27.6**
[29]	6	-	-	**61**	**44**	-	-	-	-
[2]	17	**150**	**232**	**36**	**25**	-	-	-	-
[32]	10	-	-	**54**	**34.2**	-	-	-	-
[16]	4	-	-	**37**	**32**	-	-	-	-
[10]	4	78	154	46	41	-	-	-	-
[33]	18	-	-	**55.6**	**39.8**	-	-	-	-
[34]	9	-	-	**39**	**30**	-	-	-	-
[35]	26	**136**	**174**	**49**	**38**	**24**	**38**	-	-
[15]	17	-	-	29.5	27.5	-	-	-	-

Numbers in bold are the significant difference between value before and after decompression. PaO2 = partial pressure of oxygen in arterial blood, FiO2 = fraction of inspired oxygen.
